# Training the Future Leaders in Personalized Medicine 

**DOI:** 10.3390/jpm6010001

**Published:** 2016-01-07

**Authors:** Heather Mason-Suares, David A. Sweetser, Neal I. Lindeman, Cynthia C. Morton

**Affiliations:** 1 Department of Pathology, Brigham and Women’s Hospital, Harvard Medical School, 75 Francis Street, Boston, MA 02115, USA; nlindeman@partners.org; 2 Laboratory for Molecular Medicine, Partners Personalized Medicine, 65 Landsdowne Street, Cambridge, MA 02139, USA; 3 Department of Pediatrics, Massachusetts General Hospital, 100 Charles River Plaza, Boston, MA 02114, USA; dsweetser@partners.org; 4 Department of Obstetrics, Gynecology and Reproductive Biology, Brigham and Women’s Hospital, Harvard Medical School, 75 Francis St, Boston, MA 02115, USA; cmorton@partners.org; 5 Broad Institute of Massachusetts Institute of Technology and Harvard, 415 Main Street, Cambridge, MA 02142, USA; 6 Manchester Academic Health Science Center, University of Manchester, Manchester M13 9PL, UK

**Keywords:** personalized medicine, education, genomic testing, molecular genetic pathology fellowship, clinical genetics residency, clinical molecular genetics fellowship

## Abstract

The era of personalized medicine has arrived, and with it a need for leaders in this discipline. This generation of trainees requires a cadre of new skill sets to lead the implementation of personalized medicine into mainstream healthcare. Traditional training programs no longer provide trainees with all the skills they will need to optimize implementation of this revolution now underway in medicine. Today’s trainees must manage clinical teams, act as clinical and molecular diagnostic consultants, train other healthcare professionals, teach future generations, and be knowledgeable about clinical trials to facilitate genomic-based therapies. To prepare trainees for the transition to junior faculty positions, contemporary genomic training programs must emphasize the development of these management, teaching, and clinical skills.

## 1. Introduction

The burgeoning era of personalized medicine requires dedicated future leaders with a strong foundation in advanced genomic medicine, including molecular diagnostic techniques such as next generation sequencing and whole genome/exome sequencing interpretation. These leaders must also excel at integrating personalized medicine into healthcare, and possess many additional management and teaching skills. However, few existing residency and fellowship specialties are likely to address all of these needs.

Medical specialties in advanced genomic medicine include the primary specialties of clinical genetics and clinical molecular genetics certified by the American Board of Medical Genetics and Genomics (ABMGG), and the sub-specialty in molecular genetic pathology (MGP) certified by the American Board of Pathology (ABP) and the ABMGG. Graduates of other medical specialties (Table 1), including those with more limited training in advanced genomic medicine, and are also certified to sign out genetic testing results, consideration of these skills may enhance their training as well.

**Table 1:** Medical specialties with training in genomic medicine.

Specialty	Accrediting Agencies	Primary Organization Body
Molecular Genetic Pathology	American Council of Graduate Medical Education (ACMGE)	Association for Molecular Pathology (AMP)
	American Board of Pathology (ABP)	
	American Board of Medical Genetics and Genomics (ABMGG)	
Clinical Molecular Genetics	American Board of Medical Genetics and Genomics (ABMGG)	American College of Medical Genetics and Genomics (ACMG)
Clinical Genetics	American Council of Graduate Medical Education (ACMGE)	American College of Medical Genetics and Genomics (ACMG)
	American Board of Medical Genetics and Genomics (ABMGG)	
Clinical Cytogenetics	American Board of Medical Genetics and Genomics (ABMGG)	American College of Medical Genetics and Genomics (ACMG)
Biochemical Genetics	American Board of Medical Genetics and Genomics (ABMGG)	American College of Medical Genetics and Genomics (ACMG)
Medical Biochemical Genetics	American Council of Graduate Medical Education (ACMGE)	American College of Medical Genetics and Genomics (ACMG)
	American Board of Medical Genetics and Genomics (ABMGG)	
Clinical Chemistry	American Board of Clinical Chemistry (ABCC)	American Association for Clinical Chemistry (AACC)
Clinical Pathology	American Council of Graduate Medical Education (ACMGE)	American Society for Clinical Pathology (ASCP)
	American Board of Pathology (ABP)	

The ABMGG clinical genetic residency, accredited by the American Council of Graduate Medical Education (ACGME), accepts Doctors of Medicine and Osteopathic Medicine, the majority of whom have already completed an ACGME-accredited primary residency, most commonly in pediatrics or internal medicine. This two-year program trains residents to diagnose and treat individuals with genetic disorders as well as to provide consultations for other specialists.

Directors of CLIA-certified clinical molecular diagnostic laboratories have typically completed training in either clinical molecular genetics or MGP fellowships. The clinical molecular genetic fellowship accepts Doctors of Philosophy, Medicine, and Osteopathic Medicine. This two-year program focuses on somatic and constitutional genomics. The MGP fellowship, accredited by the ACGME, is a one-year training program for Doctors of Medicine who have already achieved certification in an ACGME-accredited clinical and/or anatomic pathology residency or the ABMGG clinical genetics residency. In addition to germline and somatic testing, the MGP fellowship includes training in molecular virology and microbiology.

During these 1- to 2-year genetic specialty training programs, a trainee must transition from a novice to a competent clinician or junior laboratory director. To ensure this transition occurs, ACGME recommends competency in six areas: patient care, medical knowledge, practice-based learning and improvement, interpersonal and communication skills, professionalism, and systems-based practice [1]. These six competencies are broken down into medical genetics specialty-specific assessments, which include understanding genetic tests and results, ability to develop differential diagnoses for constitutional and somatic genetic disorders, and effective communication, management, and professional skills. ABMGG program requirements include mastering these assessments in addition to didactic sessions in advanced human and medical genetics. The following recommendations are intended to supplement training in these major programs, which provide the majority of future practitioners of advanced genomic medicine.

## 2. Above and Beyond: What It Takes to Train a Future Leader

In the new era of personalized medicine, trainees in advanced genomic medicine must develop a breadth of expertise. Trainees must be able to manage clinics or molecular diagnostic laboratories efficiently, and to build effective clinical teams including practitioners in different specialties. They must be able to work with utilization management teams to evaluate the appropriateness of tests and to develop cost-effective and accurate testing protocols. Trainees must also be prepared to educate the public and fellow medical practitioners, and to advocate on behalf of personalized medicine. Suggestions for developing these skills are provided below.

### 2.1. Advanced Management Training

Advanced management training is a necessary component of contemporary genomic training programs such as those outlined above, ensuring that trainees develop the required skills to manage their work places efficiently. Such training should minimally cover budgeting, risk management, conflict management, business plan development, and marketing as well as CLIA and FDA regulations for laboratory fellows. Training sessions should include both formal lectures, to help trainees understand basic terminology and principles, and stimulation workshops in which trainees may develop practical, hands-on experience. After completion of these minimal requirements, trainees should be involved in relevant meetings and discussions concerning these topics, and managerial functions should be incorporated into a trainee’s day-to-day clinic or laboratory responsibilities. These functions may include overseeing development of a business plan or marketing strategy with guidance and mentorship from senior faculty.

### 2.2. Building and Leading Clinical Teams

Medical geneticists are responsible for coordinating and directing multidisciplinary care for patients with genetic syndromes. Trainees must therefore develop the ability to build and manage multidisciplinary clinical teams. They must learn to work with other practitioners to integrate the expertise of the broader medical community. For example, medical geneticists may need to collaborate with general practitioners and pediatricians to improve detection and recognition of genetic and metabolic disorders, with laboratory directors to improve test utilization and interpretation, and with bioinformaticians and researchers to assist in translation of genomic medicine into clinical practice. Trainees must also be taught to work with medical staff to ensure smooth operation of the clinic, as the standard work flow for an outpatient consultation may depend upon the contributions of patient service coordinators, medical assistants, phlebotomists, laboratory staff, and genetic counselors. Trainees must also become skilled at effectively managing subspecialty clinics with unique expertise and workflows.

Training programs are obliged to provide trainees experience-building opportunities to perform these different roles. For example, trainees should participate in regular team meetings held to assess performance, evaluate patient feedback, and solicit suggestions for improvement. As trainees advance they should be required to manage these meetings, with appropriate supervision. Training programs should provide opportunities both in general genetics and in subspecialty clinics. Trainees should also receive guidance on giving appropriate praise, in providing constructive criticism, and in conflict management.

### 2.3. Laboratory-Based Clinical Consultation

Medical geneticists and molecular diagnostic directors are responsible for working with providers to request appropriate genetic testing, including the selection and succession of tests. For example, clinical geneticists or laboratorians may be incorporated into utilization management teams, which are increasingly employed to reduce costs of tests in the complex reimbursement environments of medicine today. In this role, these professionals can provide expertise in evaluating the appropriateness of requested tests and in assisting in selection of cost-effective and accurate tests. Trainees should be included in discussions with providers on strategies for reducing costs and educated on how reimbursement policies may affect these ordering strategies.

Medical geneticists and molecular diagnostic directors may be involved in contract negotiations with external testing laboratories to ensure testing agreements do not sacrifice clinical utility. Trainees must know the relative clinical utility of tests offered by external laboratories and should be involved in such contract negotiations as an aspect of their educational experience.

Medical geneticists and molecular diagnostics directors often lead genomic committees, such as Genomics Boards, that discuss the appropriateness of patients for whole-genome or -exome sequencing or review the results of genomic analyses. These meetings often involve bioinformaticians to help interpret genomic data and specialists to determine the potential clinical significance of identified sequence variants in the context of a patient’s phenotype. Such meetings can help develop treatment plans or plans for further evaluations and initiate collaborations with researchers to evaluate the functional significance of prioritized identified sequence variants. Trainees should attend such meetings, and as they become more experienced, should be required to lead meetings, with appropriate supervision.

Molecular diagnostic directors are also responsible for providing accurate and comprehensible test results. Busy practitioners and patients may improperly interpret and act upon poorly reported genetic test results. The implications of this can be profound. An insufficient result communicated poorly in a written report may result in misleading genetic or prenatal counseling, withholding of a potentially effective treatment, or inappropriate treatment with a futile targeted therapy. Trainees should therefore be able to distill and clearly communicate essential clinical information for each case. This practical skill can only be learned by communicating results to patients, families and other providers, and should be a major component of any training program.

### 2.4. Clinical Trials

As the future of personalized therapies and preventive medicine unfolds, there will be an accompanying need for healthcare professionals trained in clinical trial design and implementation. Medical geneticists may develop a niche expertise in the fundamentals of clinical trials of novel therapeutics including gene therapies employing gene editing, and involving multiple specialties as has been their role historically. Various educational opportunities are already available widely through programs funded by the National Institutes of Health Clinical and Translational Science Award (CTSA) programs. For example, the CTSA at Harvard Medical School, known as Harvard Catalyst, is dedicated to improving health by enabling collaboration and by providing tools, training, and technologies to clinical and translational investigators. Harvard Catalyst offers a course in Clinical Trial Design that is a combination of online and in-person course work over 12 weeks. Participants receive instruction in the basics of clinical trial design including how to develop a protocol, information on IRB and regulatory topics and on trial implementation. Additional topics covered include statistical analysis, budgeting, and data management. Other programs through the NIH CTSAs provide skills for trainees embarking on careers in translational research that lead to first-in-human trials. Senior fellows and junior faculty may pursue further education through NIH-funded KL2 awards that provide two years of advanced training in clinical and translational research and provide the basis for independent NIH awards (e.g., K23, K08 or R01). Through faculty mentoring, trainees should be exposed to these educational opportunities that will prepare them to become leaders in accelerating the future of personalized medicine through clinical trials.

### 2.5. Trainees Becoming the Teachers: Educating Other Healthcare Professionals

Personalized medicine’s promise to improve health care outcomes requires proper genomics education of all providers, regardless of specialty. Medical education should be enhanced with additional training in genetics and genomics, including the application of genetics and genomics to patient care. But efforts to educate practicing healthcare providers are also necessary. In a survey of over 200 internists, approximately 80% acknowledged that they required additional training in genetics. Nearly three-quarters of the internists described their knowledge of genetics as very poor or somewhat poor [2].

Our future graduates must develop effective communication skills for training current and future healthcare providers. The Harvard Medical School (HMS) Genetics Training Program develops these skills through one or more one-on-one training sessions and formal class lectures lead by current trainees. During these sessions and lectures, residents or fellows in genetic specialties train students in other medical specialties, such as residents in infectious disease, neurology, cardiology, and surgery. Additional opportunities are provided to trainees to participate through national organizations such as the American Society of Human Genetics (ASHG), whose strategic plan embraces training of healthcare professionals in genetic medicine.

### 2.6. Trainees Becoming the Teachers: Educating Lay People

Taking personalized medicine mainstream requires addressing lay misperceptions and misunderstandings of personalized medicine. The general public displays great variability in their knowledge of genetics [3,4] and lay public self-confidence is often unfounded. For example, in a survey, a majority of the public misinterpreted direct-to-consumer test results while describing the results as easy to interpret [5].

Future graduates must ensure that the general public comprehends personalized medicine and the implications of personalized medicine for mainstream health care. Therefore, trainees must learn to teach lay persons effectively about genetics and genomics. Minimally, trainees need to learn how to develop content effectively for patient educational materials, such as pamphlets or booklets. Training programs may foster more advanced skills by requiring trainees to create mass media in the form of mainstream articles or videos targeting lay audiences as well as lecturing, leading discussions, or even giving TED-style talks to the general public at forums such as science museums or coffee houses. Increasingly, unique patient groups are organizing into support groups using social media. Trainees may desire exposure and instruction in the use of social media such as Facebook and Twitter to reach lay audiences. Such sites present valuable opportunities for health care professionals to learn from patients as well as to disseminate information.

## 3. Building and Retaining Genomics Interest in Future Generations

The future of personalized medicine requires making genetic specialties attractive to upcoming medical school graduates. While genetic subspecialties strive to recruit top talent, the clinical genetics residency has not been able to fill all available residency positions nationally in the past five years [6]. What will help to fill these positions? Current clinical genetics residents have credited their decision to enter a clinical genetics residency to previous mentoring by a geneticist (50%), research in genetics (35%), or a medical school genetics course (33%) [7]. In the same survey, a large majority of graduates from US medical schools disagreed with the statement that “[m]y medical school experience provided me with enough insight into what a medical geneticist does to make an informed decision about medical genetics as a career” [7]. These results suggest a need for increased awareness not only of the field but of the role of a medical geneticist as a healthcare provider with additional mentoring available to those interested.

Professional organizations are also working to enhance interest in advanced genomic medicine. The ASHG offers career sessions and trainee workshops, events highlighted as a trainee track at all ASHG conferences. ACMG offers Medical Student Interest Groups and a Summer Genetics Scholars Program (SGSP). The SGSP is a six-week program funded by the ACMG Foundation that occurs between the first and second years of medical school. During that summer, a medical student works under the direct supervision of an experienced medical geneticist at one of over 20 institutions such as Boston Children’s Hospital, Johns Hopkins School of Medicine, or Case Western Reserve University/University Hospitals of Cleveland among others.

In the long run, developing interest in genomics requires strategies that reach far beyond medical school. Outreach programs to develop interest in genomics should be developed for primary and secondary schools. Over time, these programs may build the interest of future trainees. For example, national DNA Day, organized by the National Human Genome Research Institute (NHGRI) in conjunction with ASHG and the European Society of Human Genetics every April, offers events for primary and secondary students, teachers, and the general public to learn more about the latest research and how that knowledge could enhance their health. The Explorer Genetics program, organized by Partners Personalized Medicine every summer, is a weeklong event offered to rising senior high school students, which gives them hands-on exposure to genomic research, genetic counseling, and personalized medicine.

## 4. Future Recommendations

### 4.1. Joint Training

Many benefits follow from integrating genetics specialty training with training programs for other specialties. Such joint training increases communication between specialists, conditions specialists to work together, and combats the “siloed” nature of laboratory diagnostic practice. Trainees from different specialties rotate through labs and clinics, sharing teaching activities, research projects, and small group workshops. Such shared clinical services and training programs afford a level of interaction unmatched by conferences, grand rounds, and seminars, though these additional interactional forums are also important. For example, at Partners Healthcare affiliated hospitals and laboratories, infectious disease, neurology, and cardiology residents rotate through molecular diagnostic labs while molecular genetics fellows rotate through clinics. This integration results in clinicians better equipped to use the laboratory, and laboratorians better equipped to optimize lab preferences and communications for clinical care.

### 4.2. The Individual Development Plan

Clear, individualized training plans, such as the Individual Development Plans (IDP) [8], provide trainees a structure for achieving their career goals. This structure may help trainees advance to junior faculty. The IDP process includes four steps: self-assessment; determination of long-term career goals; creation of an IDP with assistance from a mentor; and implementation and potentially revision of the IDP. The NIH has recommended incorporating IDPs into training [9], and the HMS Genetics Training Program has already implemented an “Individualized Learning Plan” pilot program for a subset of our trainees. Through this program, trainees develop, with guidance from mentors, an “Individualize Learning Plan” including annual goals. At an annual review, trainees receive oral and written feedback from a mentor on their progress in achieving these goals.

## 5. Conclusions

Optimal implementation of personalized medicine calls for the leaders in this field to have a mastery of skills not previously required. Today’s training programs must help prepare trainees to meet those needs. Following these recommendations, in addition to the requirements of the accrediting agencies, will aid in preparing trainees to become the future leaders in personalized medicine. 
